# Knockout of the lignin pathway gene *BnF5H* decreases the S/G lignin compositional ratio and improves *Sclerotinia sclerotiorum* resistance in *Brassica napus*

**DOI:** 10.1111/pce.14208

**Published:** 2021-12-01

**Authors:** Yanru Cao, Xingying Yan, Shuyao Ran, John Ralph, Rebecca A. Smith, Xueping Chen, Cunmin Qu, Jiana Li, Liezhao Liu

**Affiliations:** ^1^ College of Agronomy and Biotechnology, Academy of Agricultural Sciences Southwest University Chongqing China; ^2^ Department of Biochemistry and the D.O.E. Great Lakes Bioenergy Research Center Wisconsin Energy Institute, University of Wisconsin Madison Wisconsin USA

**Keywords:** *Brassica napus*, Ferulate‐5‐hydroxylase, Lignin monomer, NMR, *Sclerotinia sclerotiorum*

## Abstract

Ferulate‐5‐hydroxylase is a key enzyme involved in the conversion of the guaiacyl monolignol to the syringyl monolignol in angiosperms. The monolignol ratio has been proposed to affect biomass recalcitrance and the resistance to plant disease. Stem rot caused by the fungus *Sclerotinia sclerotiorum* in *Brassica napus* causes severe losses in its production. To date, there is no information about the effect of the lignin monomer ratio on the resistance to *S. sclerotiorum* in *B. napus*. Four dominantly expressed ferulate‐5‐hydroxylase genes were concertedly knocked out by CRISPR/Cas9 in *B. napus*, and three mutant lines were generated. The S/G lignin compositional ratio was decreased compared to that of the wild type based on the results of Mӓule staining and 2D‐NMR profiling in KO‐7. The resistance to *S. sclerotiorum* in stems and leaves increased for the three *f5h* mutant lines compared with WT. Furthermore, we found that the stem strength of *f5h* mutant lines was significantly increased. Overall, we demonstrate for the first time that decreasing the S/G ratio by knocking out of the *F5H* gene improves *S. sclerotiorum* resistance in *B. napus* and increases stem strength.

## Abstract 2

The oilseed rape production is unremittingly affected by *Sclerotinia sclerotiorum*, which causes stem and leaf rot and reduces the production of rapeseed oil. Lignin is an established physical barrier against pathogens, and the different lignin monomer G and S content can have different lignin structures. In this study, four dominantly expressed *BnF5H* genes were knocked out by CRISPR/Cas9 simultaneously in *B. napus*, and the *f5h* mutants were generated. The S‐/G‐lignin composition ratio was decreased compared to that of the wild type (WT) based on the results of Mӓule staining and 2D‐NMR profiling in the KO‐7 line. The resistance to *S. sclerotiorum* in stems and leaves increased in knock‐out lines. Furthermore, we found that the stem strength of mutants was significantly increased compared to that of the WT. Collectively, for the first time, we demonstrate that knockout of the lignin pathway gene *F5H* decreases the S/G ratio, improves *S. sclerotiorum* resistance in *B. napus*, and increases stem strength.

## 1 INTRODUCTION

Oilseed rape (*Brassica napus*), a member of the *Brassicaceae* family and one of the most important oilseed crops, provides us with healthy vegetable oil suitable for cooking. However, oilseed rape production is unremittingly affected by *Sclerotinia sclerotiorum* (*S. sclerotiorum*), which causes stem and leaf rot and reduces the production of rapeseed oil by approximately 10–20% every year; in some cases, the field productivity was reduced by 80% (Adams & Ayers, 1979). *S. sclerotiorum* has long been considered to be a ubiquitous necrotrophic plant pathogenic fungus. This pathogen has a broad host range, causing disease in more than 400 plant species (Boland & Hall, 1994; Bolton, Thomma, & Nelson, 2006). *S. sclerotiorum* produces cellulase, pectinase, and cutinase after infection of the host, decomposing cell wall polymers and disrupting the structural integrity of the wall (Lumsden, 1976; Riou, Freyssinet, & Fevre, 1991). *S. sclerotiorum* secretes oxalic acid, phospholipase, and proteolytic enzymes to weaken the host's defence and provide rich nutrients for itself when it invades the plant (Collmer & Keen, 1986). Lignin is an established physical barrier against pathogens (Cesarino, 2019; Malinovsky, Fangel, & Willats, 2014).

Lignin is an amorphous phenolic heteropolymer resulting from the oxidative combinatorial coupling of 4‐hydroxyphenylpropanoids. Lignin deposition can strengthen and thicken the secondary cell wall, which is crucial for the upright growth of terrestrial plants and adaptation to the terrestrial environment (Carrier et al., 2011; Kenrick & Crane, 1997; Zhong & Ye, 2015). The deposited lignin is mainly distributed in vessel, tracheid, interfascicular fibre, and thick‐walled extracellular components (Kenrick & Crane, 1997; Zhong & Ye, 2015). It is a major component of secondary cell walls and plays an important role in plant growth and development, as well as in the defence responses to various pathogens (Dixon, 2001; Dixon et al., 2002; Naoumkina et al., 2010; Zhao & Dixon, 2014). The lignin content approaches that of cellulose in vascular plants, encrusting the polysaccharides (cellulose and hemicelluloses) and perhaps connecting to hemicelluloses; in grasses, covalent bonding of arabinoxylan hemicellulosic polysaccharides to lignin mediated by ferulate is well established (Ralph, 2010). The interaction not only enhances the mechanical strength of plants and prevents cell wall collapse but also prevents toxins in the pathogen from penetrating into the host that would otherwise allow nutrients from the host to be utilized by the pathogen (Boerjan, Ralph, & Baucher, 2003; Ride, 1978; Weng & Chapple, 2010).

The complexity of lignin arises both from the relative proportion of the three major monomeric units from which it primarily derives, and the nature of the various dimeric units that are described by their characteristic inter‐unit chemical bonding. The lignin monomer ratio has been identified as an important structural factor affecting biomass recalcitrance (Holwerda et al., 2019; Sakamoto et al., 2020; Yang et al., 2019). Lignin synthesized via the phenylpropanoid pathway initiating from phenylalanine and tyrosine, the three major monolignols, which differ in their methoxylation degrees: no methoxyls in *p*‐coumaryl alcohol, one in coniferyl alcohol and two in sinapyl alcohol. These monolignols are synthesized in the cytoplasm and diffuse or are transported to the cell wall where they are polymerized into lignin, creating the *p*‐hydroxyphenyl (H), guaiacyl (G) and syringyl (S) units (Boerjan et al., 2003; Ko, Ximenes, Kim, & Ladisch, 2015; Ralph et al., 2004). These lignin units are present at different levels, and their types vary substantially among different plant species (Donaldson, 2001). The syringyl (S) monolignol has a methoxylated C‐5 position, whereas the guaiacyl (G) monolignol is unsubstituted at the C‐5, allowing it to bond with other monomers/units through carbon–carbon bonds and therefore forming a more condensed polymer (Tobimatsu et al., 2013).

Researchers have found that lignin composition may affect disease resistance in plants. Silencing cinnamyl alcohol dehydrogenase (CAD), caffeic acid *O*‐methyltransferase (COMT) or caffeoyl‐CoA *O*‐methyltransferase (CCoAOMT) in diploid wheat caused higher penetration of *Blumeria graminis*, which revealed the importance of G monolignol biosynthesis in defence against pathogen invasion (Bhuiyan, Gopalan, Yangdou, & John, 2009). Increased deposition of the more condensed G‐lignin after inoculation with *S. sclerotiorum* may present an effective way to prevent pathogen ingress into the vascular system (Eynck, Séguin‐Swartz, Clarke, & Parkin, 2012).

Ferulate‐5‐hydroxylase (F5H) catalyses the key step in syringyl lignin biosynthesis. F5H, a cytochrome P450‐dependent monooxygenase of the CYP84 subfamily, catalyses the hydroxylation of G‐lignin monomer precursors in the phenylpropanoid pathway to synthesize the S‐lignin monomer, sinapyl alcohol. The Arabidopsis *f5h* mutant contains less S‐type lignin units and instead produces G‐rich lignin, as in gymnosperms. Overexpression of miR6443 decreased the transcript level of *F5H2*, resulting in a significant reduction in the S‐lignin content in transgenic plants (Fan et al., 2020). Overexpression of *F5H* in Arabidopsis deposited higher sinapyl alcohol‐derived S‐units compared to wild‐type (WT) plants, and, under the control of the specific cinnamate‐4‐hydroxylase (*C4H*) promoter, the transgenic plants had lignins composed almost entirely of S‐type lignin units (Chapple, Vogt, Ellis, & Somerville, 1992; Meyer, Shirley, Cusumano, Belllelong, & Chapple, 1998; Stewart, Akiyama, Chapple, Ralph, & Mansfield, 2009). Compared to the WT and *f5h* mutant, the *F5H*‐overexpressing transgenic line significantly reduced the stiffness of the cell walls in the region of the compound middle lamella as determined by contact resonance force microscopy (Ciesielski, 2014). Overexpression of *F5H* also resulted in a significant increase in the S/G lignin ratio in various plant species, such as poplar, alfalfa and tobacco (Franke et al., 2000; Marita, Ralph, Hatfield, & Chapple, 1999; Reddy et al., 2005; Shuai et al., 2016).

To date, there are no reports on the effect of the lignin monomer ratio on resistance to *S. sclerotiorum* in *B. napus* or other Brassica species. Our strategy was therefore to use genetic engineering to target lignin composition and examine the development of resistance to recalcitrant diseases, for example the white mould caused by the fungal pathogen *S. sclerotiorum*. In this study, to probe the effect of lignin composition and resistance to *S. sclerotiorum* in oilseed rape, *BnF5H* was knocked out by CRISPR/Cas9 in *B. napus* and inoculated with *S. sclerotiorum*, after which the lignin compositions in the plants were profiled. Notably, our data demonstrated that decreases in the S/G lignin compositional ratio can improve the resistance to *S. sclerotiorum in* oilseed rape.

## 2 RESULTS

### 2.1 Genome‐wide analysis of the *BnF5H* gene family in *B. napus*

**Figure 1:**
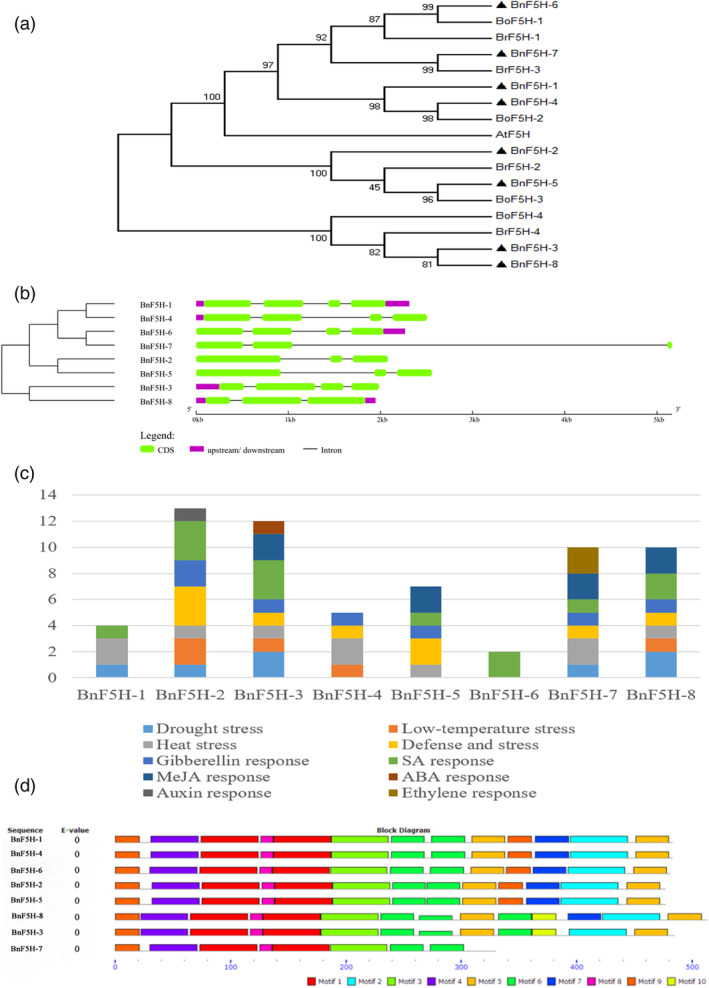
Genome‐wide analysis of the *BnF5H* gene family in *B. napus*. (a) Phylogenetic analysis of BnF5H proteins from *A. thaliana*, *B. rape*, *B. oleracea*, and *B. napus*. The protein sequences of eight BnF5Hs with one AtF5H, two BoF5Hs, and two BrF5Hs were used to construct the NJ tree with 1,000 bootstraps, designated Group I, Group II and Group III. (b) The exon–intron structure of the *BnF5H* genes according to their phylogenetic relationship. (c) The 1,500 bp sequence upstream of the transcriptional start codons was used to analyse cis‐regulatory elements using the PLACE database. (d) The conserved motifs of the BnF5H proteins presented according to their phylogenetic relationships. These motifs were identified by MEME, and boxes of different colours represent different motifs [Colour figure can be viewed at wileyonlinelibrary.com]

The *BnF5H* gene family was clustered into three clades, designated Type I, Type II and Type III. *BnF5H‐1*, *BnF5H‐4*, *BnF5H‐6* and *BnF5H‐7* belong to Type I; *BnF5H‐2* and *BnF5H‐5* belong to Type II; and *BnF5H‐3* and *BnF5H‐8* belong to Type III (Figure 1a). *BnF5H* grouping into the same branch may have similar protein affinities.

All of the Group I members, *BnF5H‐1*, *BnF5H‐4*, and *BnF5H‐6*, are composed of four exons and three introns, and *BnF5H‐7* consists of three exons and two introns. Group II members, *BnF5H‐2* and *BnF5H‐5*, are composed of three exons and two introns, and their genetic structures are similar. In Group III, *BnF5H‐3* is composed of four exons and three introns, and *BnF5H‐8* is composed of three exons and two introns. Although there are differences in the number of exons and introns, the general structures are similar (Figure 1b). These results indicated that members within a single branch had highly similar gene structures, which produced proteins with highly similar functions.

Cis‐elements, binding sites for transcription factors, are particularly important in the regulation of gene expression (Liu et al., 2014). To probe the function and potential regulatory mechanisms of *BnF5H*, the 1,500 bp sequence upstream of the transcriptional start codons was used to analyse cis‐regulatory elements using the PLACE database. A total of 10 components are potentially responsive to stress and hormones, including LTR (temperature‐responsive element), MBS (involved in drought inducibility), defence and stress‐responsive elements (TC‐rich repeats), HSE (heat stress), GARE motif (gibberellin response element), TCA element (responsive to salicylic acid), CGTCA motif (MeJA response), ABRE (ABA response element), TGA element (responding to auxin) and ERE (ethylene response) (Figure 1c). These cis‐elements suggested that *BnF5H* is likely to be involved in heat stress, salicylic acid and defence to stress responses.

Ten conserved motifs were recognized: motifs 1–9 were present in Group I and Group II branches (*BnF5H‐1*, *BnF5H‐2*, *BnF5H‐4*, *BnF5H‐5*, *BnF5H‐6*), whereas motif 10 was present in Group III (*BnF5H*‐3, *BnF5H*‐8) (Figure 1d). The results indicated that a single branch had similar motifs, which is consistent with their functions.

### 2.2 Expression profiles of *BnF5H* in *B. napus*

**Figure 2:**
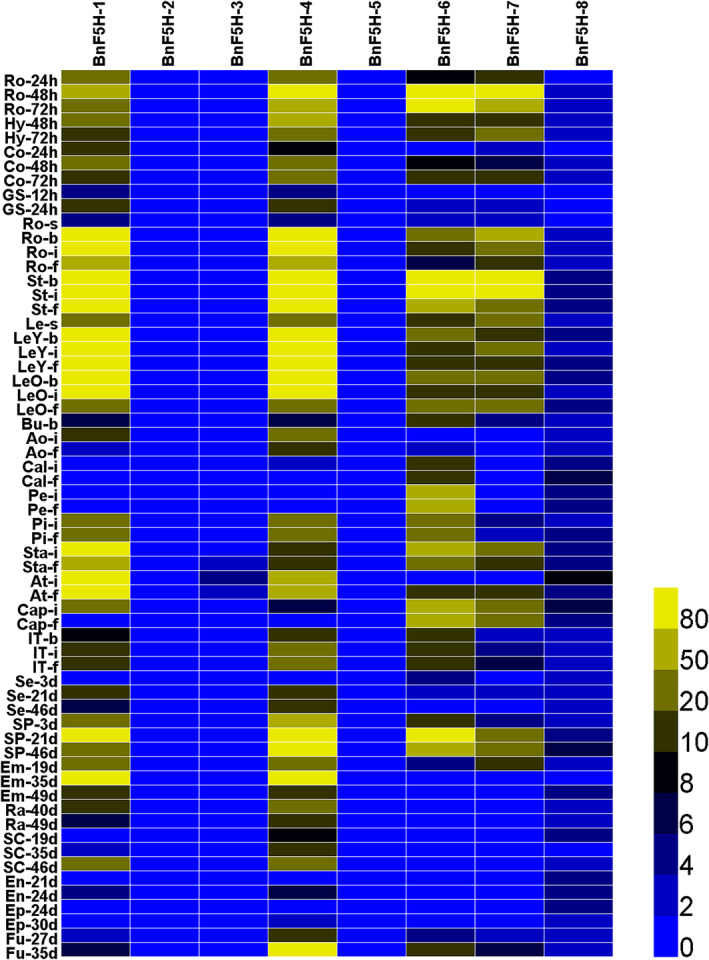
Expression levels of *BnF5H* genes in different tissues at different stages of *B. napus*. RNA‐seq datasets from *B. napus* ZS11 analyses of *BnF5H* expression in 63 different tissues and organs of *B. napus* at different developmental stages. The 24, 48 and 72 h labels indicate the time that passed after seed germination. The 3, 19, 21, 24, 27, 30, 35, 40, 46 and 49 days labels indicate the number of days that passed after the flowering stage. The bar in the lower right corner represents fragments per kilobase of exon per million reads mapped (FPKM) values, and different colours represent different expression levels. Ao, anthocaulus; At, anther; b, bud stage; Bu, buds; Cal, calyx; Cap, capillament; Co, cotyledon; Em, embryo; En, endopleura; Ep, exocarp; f, full‐bloom stage; Fu, funicle; GS, germinate seeds; Hy, hypocotyl; i, initial flowering stage; IT, inflorescences top; Le, leaf; Pe, petal; Pi, pistil; Ro, root; s, seedling stage; SC, seed coat; Se, seed; SP, silique; St, stem; Sta, stamen [Colour figure can be viewed at wileyonlinelibrary.com]

The expression profiles of *BnF5H* in various tissues showed that *BnF5H‐1*, *BnF5H‐4*, *BnF5H‐6*, and *BnF5H‐7* are the dominant genes expressed in the *BnF5H* gene family. They are expressed in roots, stems, leaves and pods and highly expressed in hypocotyls, cotyledons, buds, flowers and seeds (Figure 2). The expression patterns of these four *BnF5Hs* suggested that they play an important role in regulating the *B. napus* lignin processes, and their similar expression was coinciding with their cluster and gene structure. In contrast, the expression of the other four *BnF5Hs* was extremely low in the tested tissues (Figure 2).

### 2.3 Subcellular localization

**Figure 3:**
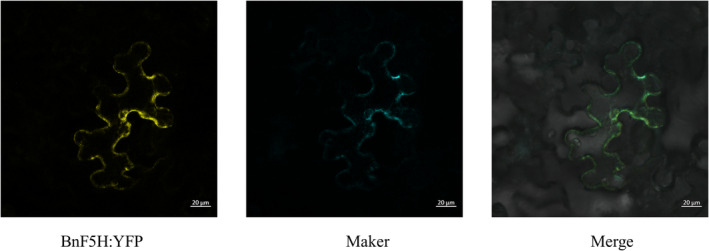
Subcellular localization of BnF5H‐1 protein. *In planta* localization in *Nicotiana benthamiana* leaves of yellow fluorescent protein (YFP), membrane marker cyan fluorescent protein (CFP) and merged fluorescence from CFP and YFP [Colour figure can be viewed at wileyonlinelibrary.com]

The subcellular localization prediction of BnF5H family member proteins suggested that BnF5H might be a membrane‐localized protein. To confirm the online prediction, the subcellular localization of BnF5H was carried out by transient expression in tobacco epidermal cells. Yellow fluorescence was exclusively observed in the plasma membrane of tobacco epidermal cells when the recombinant construct was used, indicating that BnF5H‐1 was specifically localized to the plasma membrane (Figure 3).

### 2.4 CRISPR/Cas9‐mediated mutations of *BnF5H* in *B. napus*

**Figure 4:**
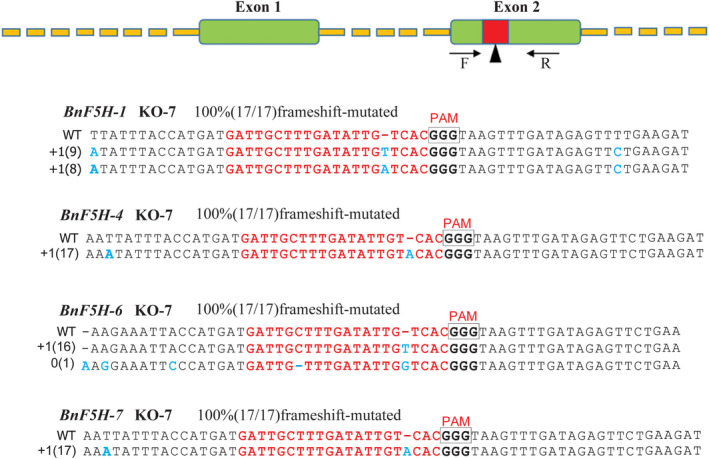
Cas9‐sgRNA generated heritable mutations in the *BnF5H* gene in *B. napus*. Targeted mutagenesis of *F5H* in *B. napus* is identified by sequencing KO‐7 plants. The CRISPR/Cas9 targeted sequences in the region of exon 2 of *BnF5H* are indicated in the red box. The numbers in the left‐most column prefixed with “−” and “+” show how many nucleotides are deleted or inserted, and numbers in parentheses represent the number of detected mutations with such mutant alleles [Colour figure can be viewed at wileyonlinelibrary.com]

One 20‐bp sequence followed by a trinucleotide (5′‐NGG‐3′) protospacer adjacent motif (PAM) located in the second exon region of *BnF5H* was selected as the sgRNA complementary site (Figure 4). To screen the targeted mutagenesis of *BnF5H*, genomic DNA from 10 *T*_0_ plants harbouring the Cas9‐sgRNA construct was extracted for PCR amplification and sequencing. The PCR‐amplified products were detected by sequencing analysis of five randomly selected clones from individual transgenic plants. The results revealed that there were insertions (+) or deletions (−) at the desired target sites caused by the CRISPR/Cas9 system, introducing InDels into the *BnF5H* gene via the nonhomologous end‐joining (NHEJ) repair pathway. Among them, the sequence result of transgenic plants indicated that the desired *BnF5H‐1*, *BnF5H‐4*, *BnF5H‐6*, and *BnF5H‐7* were simultaneously mutated at the target sites or nearby, which included base mutations, deletions or insertions, and we named those lines by KO‐7, KO‐8 and KO‐10 (Figures 4, S1, and S2). To continue screening for inherited targeted mutagenesis in this progenies, 17 *T*_1_ plants from KO‐7, KO‐8, and KO‐10 were randomly selected for sequencing, and all the plants showed the same mutagenesis. The *T*_2_ plants were analysed for the same mutations by sequencing before the lignin chemical composition and inoculation tests (Figures S3 and S4). The results indicated that Cas9‐sgRNA successfully generated three *f5h* mutants in the *BnF5H* gene, which can be inherited in *B. napus* offspring.

### 2.5 Histochemical studies

**Figure 5:**
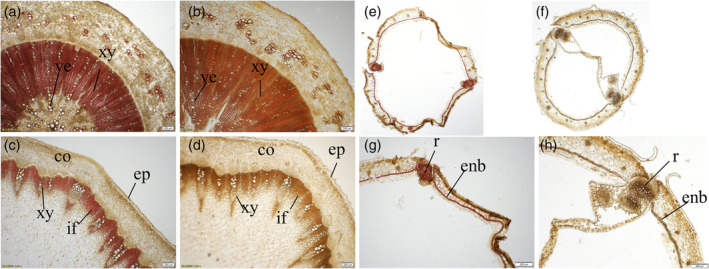
Mäule staining cross sections of WT and KO‐7 plants. (a, b) Mӓule reagent staining of roots at the initial flowering stage for the WT (left) and KO‐7 (right) plants. (c, d) Mӓule reagent staining of stems at the initial flowering stage for the WT (left) and KO‐7 (right) plants. (e, f) Mӓule reagent staining of the siliques at 40 days after flowering for the WT (left) and KO‐7 (right) plants. (g, h) are larger versions of (e, f). co, cortex; enb, endocarp b; ep, epidermis; if, interfascicular fibre; r, replum; ve, vessel; xy, xylem [Colour figure can be viewed at wileyonlinelibrary.com]

To verify whether the lignin composition changed as a result of knocking out the lignin pathway gene *BnF5H*, stem, root, and silique lignin compositions from *T*_2_ plants of *f5h* mutant KO‐7 were analysed by microscopy after staining. With Mӓule histochemical staining, the lignified tissues stained red if they contained S‐units and brown if only G‐units were present (Goujon, Sibout, Eudes, Mackay, & Jouanin, 2003). *B. napus*, as an angiosperm, mainly includes G‐ and S‐lignin and only a small amount of H lignin. As is normal in dicots, G‐lignin was detected in the walls of xylem vessels, and S‐lignin was detected as a major constituent of the walls of fibres in the stems and roots of WT plants. The Mӓule reagent staining at the initial flowering stage of stem and roots showed that the xylem stained red in the WT (Figures 5a,c), whereas the xylem in the *f5h* mutant plants stained brown (Figure 5b,d). The WT siliques displayed a red colour in the lignified region of replum and endocarp b (Figure 5e,g), whereas the *f5h* mutant plants stained with a tan colour in the same region of the silique (Figure 5f,h). The significant difference in Mӓule staining in lignin‐rich tissues indicates the differential lignin monomer composition in WT versus *f5h* mutant plants, which established that we had successfully knocked out the four dominantly expressed *BnF5H* genes by CRISPR/Cas9 in *B. napus*.

### 2.6 Determination of lignin content and composition

**Figure 6:**
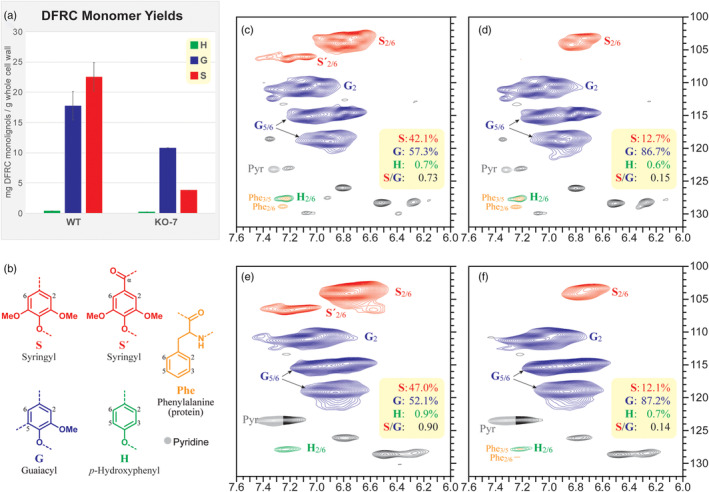
Determination of lignin content and composition. (a) Monolignols released by DFRC from WT and KO‐7. (b) Different monomer units by different colour. (c, d) 2D‐HSQC‐NMR of WT and KO‐7 enzyme lignin samples. (e, f) 2D HSQC‐NMR of WT and KO‐7 whole cell wall samples. S, G and H units are colour‐coded with their matching structures, below. Relative levels are from volume‐integration of contour peaks [Colour figure can be viewed at wileyonlinelibrary.com]

To quantitatively assess changes in lignin composition, derivatization followed by reductive cleavage (DFRC) analysis was used. From WT samples, DFRC released (the peracetates of) sinapyl alcohol, coniferyl alcohol, and trace amounts of *p*‐coumaryl alcohol (Figure 6a,b), with sinapyl alcohol being the most prominent. Mutant *f5h* plants exhibited alterations in lignin composition and amount. The levels of monolignols released by DFRC from *f5h* mutant plants were significantly lower than those released by the WT (Figure 6a and Table S2), implying either a lower lignin content in *f5h* mutant plants or a more condensed lignin structure. Only monolignols that are bound by β‐*O*‐4 linkages can be released by DFRC, but a higher G‐lignin content leads to the formation of more C–C bonds in the lignin and impedes monolignol release. Coniferyl alcohol accounts for over half of the lignin monomers released by DFRC, and the amount of sinapyl alcohol was four‐ to fivefold lower in the *f5h* mutant plants (Figure 6a).

2D‐NMR analysis revealed that WT produces an approximately 1:1 ratio of S‐lignin to G‐lignin (Figure 6c,e and Table S2). *B. napus* does not naturally produce any detectable levels of monolignol conjugates or other alternative lignin monomers, such as monolignol *p*‐coumarates, monolignol ferulates, monolignol *p*‐hydroxybenzoates, or tricin. The enzyme lignin (EL) NMR spectra (Figure 6c,d) yielded the same results as the whole cell wall spectra (WCW) (Figure 6e,f), and both corroborated the results of the DFRC analysis. Mutant *f5h* plants had a much higher proportion of G‐units than S‐units (S:G:H 12.7:86.7:0.6 vs. 42.1:57.3:0.6 in WT), as was also observed by DFRC. The S/G ratio in *f5h* mutant line was reduced from 0.73 (in WT) to 0.15 (Figure 6c,d). These results demonstrate that the *BnF5H* gene has been knocked out or down‐regulated, such that the BnF5H enzyme (along with any others that might be active for 5‐hydroxylation) was not capable of synthesizing WT levels of S‐lignin in the *f5h* mutant plants.

### 2.7 Knockout of *BnF5H* increased stem strength and altered stem cell wall structure

**Figure 7:**
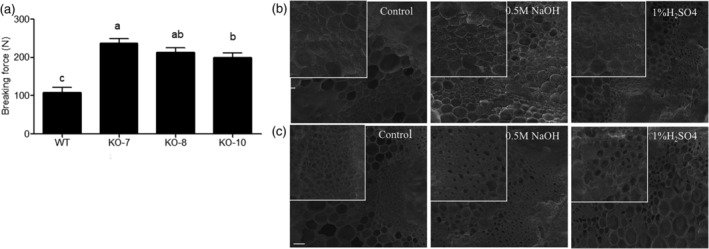
Measurement of stem breaking force and SEM image of the oilseed rape cell wall surface. (a) Stem strength of WT and mutant lines at the harvest stage. Error bars indicate ±*SD*. Significant differences were determined by one‐way ANOVA and Tukey's test, *p* < .05. (b, c) SEM image of the cell wall surface after alkali and acid pretreatment. (b), Control, after 0.5 M NaOH and 1% H_2_SO_4_ pretreatment of WT from left to right. (c), Control, after 0.5 M NaOH and 1% H_2_SO_4_ pretreatment of KO‐7 from left to right. The area in the white square highlights the structural differences between different chemical pretreatments

Stem strength is positively related to stem lodging resistance, for which it can be used as a reliable index. To determine the effect of an altered lignin monomer ratio on stem strength, the mature stems of WT and knockout plants were used to measure the breaking force. The breaking force of the *f5h* mutant plants was significantly higher than that of the WT plants in the same developmental stage, with the same diameter and position along the stem (Figure 7a and Table S3). According to our results, stem strength might be related to the S/G lignin composition ratio in the stem of *B. napus*.

The stem structure was assessed by SEM after alkaline and acidic treatment of the stem cross sections. The cells exhibited tightly packed arrangements in *f5h* mutant (Figure 7c), whereas the cells showed extensive, loosely packed arrangements in WT plants (Figure 7b). Compared to the control (without treatment), mild alkali‐treated dissection showed a similar cell wall surface, whereas the acid‐treated dissection exhibited a very rough surface (Figure 7b). The vascular bundles were smaller and more compact in *f5h* mutant plants than in WT plants in both the control and treated samples (Figure 7c).

### 2.8 Knockout of *BnF5H* shows enhanced resistance to *S. sclerotiorum*

**Figure 8:**
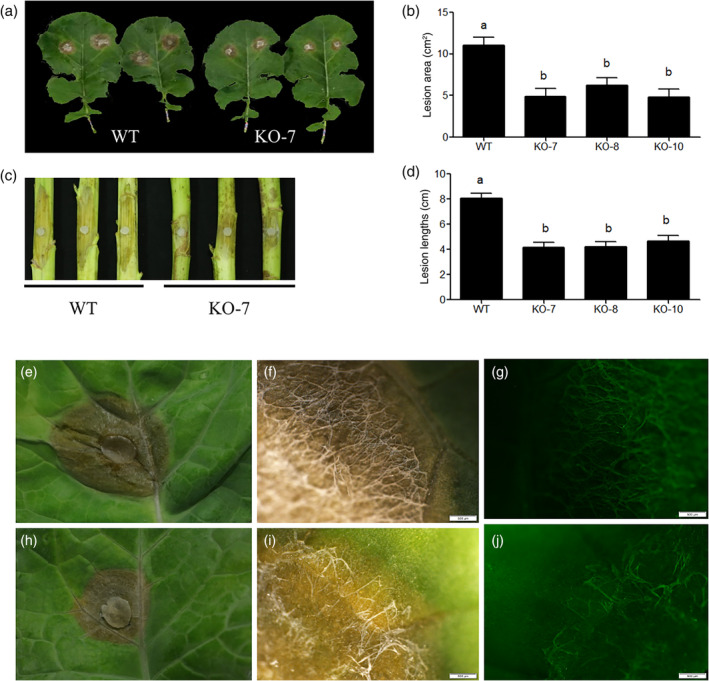
Resistance of mutant lines to *S. sclerotiorum* infections. (a) Disease responses of inoculated plants at 48 hr post‐inoculation (hpi). Photographs were taken of leaves from WT (left) and KO‐7 (right) plants. (b) The lesion area in the infected leaves from WT and mutant lines. (c) Disease responses of inoculated plants at 72 hr post‐inoculation (hpi). Photographs were taken of stems from WT (left) and KO‐7 (right) plants. (d) Lesion lengths in infected stems from WT and mutant lines. Values are means of three replications. Error bars indicate ±*SD*. Significant differences were determined by one‐way ANOVA and Tukey's test, *p* < .05. WT and KO‐7 leaves showing lesions at 24 hr post‐inoculation in (e, h), respectively. Stereomicroscope images show the differences in the growth status of mycelia on WT (f) and KO‐7 (i) leaves. Fluorescence microscopy shows the differences in the growth status of mycelia between WT (g) and KO‐7 (j) leaves. Scale bars: 500 μm for (f, i) and (g, j) [Colour figure can be viewed at wileyonlinelibrary.com]

Compared with the severe disease symptoms appeared on the WT leaves, smaller necrotic lesions were noted on the leaves of the *f5h* mutant plants (Figure 8a,b and Table S3). The stems from the mature stage of *f5h* mutant plants showed smaller lesion lengths than WT plants when the plaque area was measured after inoculation with *S. sclerotiorum* for 72 hr (Figure 8c,d and Table S3). The stem from the flowering stage inoculated with *S. sclerotiorum* gives the same results (Figure S5). The above results show that the decreased S/G ratio resulted in significantly improved resistance to *S. sclerotiorum* in *B. napus* in both stems and leaves.

Infection cushions, notable in both WT (Figure 8e) and *f5h* mutant plants (Figure 8h), showed surface hyphae emanating from the cushions and extending across the leaf and out of view. Changes in lesion size indicated the rapid expansion on WT leaves (Figure 8e,f), but less growth was evident on *f5h* mutant leaves (Figure 8h,i). Enlarged areas show the differences of lesions between WT (Figure 8g) and *f5h* mutant leaves (Figure 8j).

## 3 DISCUSSION

*S. sclerotiorum*, a soil‐borne plant pathogen, causes disease in more than 400 species of 278 genera belonging to 75 families and is widely distributed in various parts of the world (Boland & Hall, 1994; Bolton et al., 2006). In *B. napus*, crop rotation and the application of fungicides are currently the major defences against Sclerotinia to improve long‐term yield for farmers. To date, complete resistance has not been reported and researchers are trying different methods to improve the Sclerotinia resistance in *B. napus*. Overexpression of an nsLTPs‐like antimicrobial protein gene (LJAMP2) from motherwort (*Leonurus japonicus*) enhances resistance to *S. sclerotiorum* in oilseed rape (Jiang et al., 2013). Overexpression of *BnWRKY33* enhances resistance to *S. sclerotiorum* in oilseed rape (Wang et al., 2014). In our previous study, a total of 520 lines from different *B. napus* sources were used to identify resistance to *S. sclerotiorum*, and the relative susceptibility showed a significant correlation with the S/G ratio (Wei et al., 2017). We were therefore curious about the effects of lignin monomer composition on the resistance of *S. sclerotiorum*.

There are controversial reports on the influence of the lignin composition and structure on plant disease resistance. Some studies have shown that syringyl levels were positively correlated with plant disease resistance in tomato and poplar (Gayoso, Pomar, Novouzal, Merino, & De Ilarduya, 2010; Skyba, Douglas, & Mansfield, 2013). Higher G and H levels and lower S levels accumulated after soft rot disease infection in Chinese cabbage (Zhang, Yang, & Ma, 2007). Similarly, the defence response in eucalyptus induced the accumulation of G‐lignin (Hawkins & Boudet, 2003). The ratio of G/S units increased after inoculation in both resistant and susceptible inoculated tomato plants (Gayoso et al., 2010). In *Camelina sativa*, the S/G ratio in disease‐resistant plants was higher than that in susceptible plants, but the S/G ratio decreased in inoculated plants compared with mock‐inoculated plants after 7 days of inoculation for both resistant and susceptible lines (Eynck et al., 2012). Resistant cotton accumulated more G than S‐lignin and the G/S increased, but the susceptible materials behaved contrarily upon *V. dahliae* inoculation (Xu et al., 2011). In tomato, the gall treated by benzothiadiazole (priming agent in plant defence) can increase the lignin pathway gene expression and more G accumulates than S monomer (Veronico et al., 2018). The controversial reports could imply that disease resistance is a typical quantitative trait, and plants with a high G monomer content may not always show more resistance in comparison because of the genetic background difference. Also to be considered, a disease response will enhance G monomer levels upon the infection to counter against the pathogen invasion. In our study, the three *f5h* mutants with a decreased S/G ratio exhibited increased resistance to *S. sclerotiorum* in both stems and leaves compared with WT *B. napus* (Figure 8 and Table S3). Even though the leaf contains less lignin compared with stem, the lignin monomer modification still positively affects Sclerotinia resistance.

The proportion of S‐ and G‐lignin units is a crucial chemical property of lignin because the ratio of S/G lignin affects the recalcitrance of lignocellulose for industrial utilization by enzymatic hydrolysis of polysaccharides in biomass. Previous studies have reported that genetic manipulation of the biosynthetic pathway to alter the lignin polymer can reduce recalcitrance to the conversion processes in Arabidopsis (Li et al., 2010; Shi et al., 2016), poplar (Huntley, Ellis, Gilbert, Chapple, & Mansfield, 2003; Mansfield, Kang, & Chapple, 2012; Studer et al., 2011), and other hardwoods (Santos, Lee, Jameel, Chang, & Lucia, 2012). One exception reported that there was no significant correlation between saccharification efficiency and 5‐hydroxyguaiacyl lignin levels and S/G ratio (Wu et al., 2019). In our work, the genetic manipulation of the monomeric composition of the lignin polymer in *B. napus* may alter the recalcitrance of lignocellulose during enzymatic digestion, as we found the stem structure was more tightly packed in the *f5h* mutants, and therefore, saccharification efficiency needs further investigation in the future. Our main aim, however, is to improve the agronomic characteristics of *B. napus*.

Lodging is a significant problem in crop production because it causes yield loss, poor grain filling, and impedes mechanical harvesting (Berry, Sterling, Spink, Baker, & Ennos, 2004). There are two types of lodging in oilseed rape, root lodging due to root anchorage system failure and the stem lodging as the stem breaks down or buckles. Stem strength is determined by its chemical and biochemical components and their physical structure. The Arabidopsis *irx4* mutant, defective in a cinnamoyl‐CoA reductase, failed to grow upright because of the reduced lignin content (Jones, Ennos, & Turner, 2001). In Arabidopsis, the dominant repression of lignin pathway transcription factors MYB58 and MYB63 produces the pendent stem phenotype (Zhou, Lee, Zhong, & Ye, 2009). Overexpression of the wheat COMT gene leads to a higher lignin content, improved stem strength, and a lower lodging index (Ma, 2009). The nitrogen and density application can change the lignin composition and results showed that syringyl (S) monomers were the predominant lignin monomeric units responsible for enhancing mechanical strength in wheat (Luo, Ni, Pang, Jin, & Wang, 2019). In this study, the *f5h* mutants provided us with direct evidence for the stem strength dependence on lignin composition, which might be a significant finding to mitigate lodging problems in oilseed rape.

In conclusion, resistance to *S. sclerotiorum* correlated with lignin monomer composition in *B. napus f5h* mutants in which G‐units were produced and incorporated into the lignin in higher proportions than S‐units. Our findings provide new insights into the disease resistance mechanism of oilseed rape on the basis of lignin chemical composition.

## 4 MATERIALS AND METHODS

### 4.1 Plant materials and transformation

The oilseed rape line “Westar” was used as the transformation receptor, and genetic transformation was performed using Agrobacterium‐mediated methods described previously (Cardoza & Stewart, 2003), followed by hygromycin (25 mg/L) or kanamycin (50 mg/L) selection on Murashige–Skoog (MS) medium. Transgenic lines were verified for each construct by polymerase chain reaction (PCR) with gene‐specific primers. The knockout line in the *T*_2_ generation and wild‐type plants were grown in an isolated field and managed as usual.

### 4.2 Analysis of gene expression profiles

To characterize the temporal and spatial expression patterns of *BnF5H*, we analysed 63 different tissues, which included roots, stems, leaves, flowers, siliques, and seeds from the *B. napus* cultivar ZS11 at different developmental stages (germination, seedling, bud, initial flowering, and full‐bloom stages), using RNA‐seq datasets. These transcriptome sequencing datasets were conserved in BioProject ID PRJNA358784. We quantified the gene expression levels according to their fragments per kilobase of exon per million reads mapped (FPKM) values using Cufflinks with default parameters, and a heatmap was drawn using the R package.

### 4.3 Genome‐wide analysis

To elucidate the evolutionary relationships of the *BnF5H* gene family, the *BnF5H* protein sequences from *A. thaliana*, *B. napus*, *B. rapa* and *B. oleracea* were retrieved. Phylogenetic trees were constructed using MEGA 5.1. The *BnF5H* gene exon–introns were analysed by using the GSDS website to study the *BnF5H* gene structures. To identify protein motifs, we analysed the full‐length protein sequences of *BnF5H* using MEME software. The 1,500 bp sequence upstream of the transcriptional start codons was used to analyse cis‐regulatory elements using the PLACE database.

### 4.4 Subcellular localization

Subcellular localization of BnF5H family member proteins was predicted using the Psort website (http://psort.hgc.jp). To confirm the online prediction, the ORF of *BnF5H‐1* was fused to the 3′‐terminus of yellow fluorescent protein (YFP) under the control of the constitutive cauliflower mosaic virus 35S promoter (CaMV35S). Agrobacteria with OD600 of 0.8 was resuspended in infiltration solution (10 mM MgCl_2_, 10 mM MES, 0.2 mM acetosyringone) for 3–5 hr until the OD600 reaches 0.5, followed by incubation in the dark for 3 hr before coinfiltration into leaves of *N. benthamiana*. The fluorescent signal was recorded with a microscope (Leica SP8, Germany) at 48 hr after coinfiltration.

### 4.5 Generation of the *f5h* mutant by CRISPR/Cas9

The full‐length DNA sequence of the *BnF5H* gene family was screened using the Genoscope *Brassica napus Genome Browser* (http://www.genoscope.cns.fr/brassicanapus/). Cotargeting primer gRNAs (Table S1) of the dominantly expressed *BnF5H‐1*, *BnF5H‐4*, *BnF5H‐6*, and *BnF5H‐7* were designed using the CRISPR‐P 2.0 website (http://crispr.hzau.edu.cn/CRISPR2/). One putative target site located at the second exon of the *BnF5H* coding sequence was selected to design the sgRNA sequences based on their GC abundance. The sgRNA sequence was 20 bp long and was adjacent to the PAM (NGG) region, which must be located in a conserved region of the four *BnF5H* members simultaneously and within a functionally conserved domain. To ensure the specificity of the sgRNA sequence, BLAST was performed on the online website (http://brassicadb.org/brad/). Oligos were designed to specifically target *BnF5H*, and sgRNA cassettes were assembled into binary CRISPR/Cas9 vectors. Genomic DNA was isolated from the *f5h* mutant using a plant genomic DNA kit (TIANGEN), followed by PCR amplification using gene‐specific primers (Table S1). The *BnF5H‐1*, *BnF5H‐4*, *BnF5H‐6*, and *BnF5H‐7* PCR products were cloned into the PEASTY‐T_1_ vector (TransGen Biotech), and at least 20 clones for each mutant line were randomly selected for sequencing.

### 4.6 Histochemical analysis

Mäule histochemical staining was used to survey and evaluate lignin monomer composition. The stems and roots of *f5h* mutant and WT plants were sampled at the initial flowering stage, and the silique was sampled 40 days after flowering. The cross section was stained by Mäule reagent (Chapple et al., 1992; Chen et al., 2002). The sections were treated with 1% KMnO_4_ for 5 min, rinsed with water, treated with 3% HCl for 2 min, rinsed again with water, and mounted in concentrated 29% NH_4_OH for examination on an OLYMPUS MUX10 microscope.

### 4.7 Determination of lignin content

The intact stem parts of the WT and KO‐7 plants were used to determine lignin content after inoculation, as previously described (Mansfield et al., 2012). The ground samples were solvent‐extracted with sequential extractions of water (3 × 45 ml), 80% ethanol (3 × 45 ml) and acetone (1 × 45 ml). Each extraction consisted of a 20‐min sonication step followed by a 20‐min centrifugation step. The extract‐free samples were dried completely under vacuum. The samples were then ball‐milled using a Fritsch pulverisette 7 mill. EL was generated from ball‐milled material by treating the samples with cellulase enzyme (Cellulysin, MilliporeSigma‐Calbiochem) in acetate buffer (pH 5.0). Samples were incubated at 35°C for 72 hr, followed by two extractions with acetate buffer, 20 min of sonication and 20 min of centrifugation. More cellulase enzyme was added to fresh acetate buffer, pH 5.0, and incubated at 35°C for 72 hr. Enzyme lignin samples were extracted with distilled water and then freeze‐dried to obtain the final enzyme lignin (with a low polysaccharide content).

DFRC analysis was performed as described in the optimized DFRC protocol (Regner, Bartuce, Padmakshan, Ralph, & Karlen, 2018). Two technical and three biological replicates were analysed per sample, with 50 mg of ground and solvent‐extracted sample used per technical replicate. The internal standard mix consisted of G‐d_8_, S‐d_8_, S‐DD*p*CA‐d_10_, and S‐DDFA‐d_10_. Samples were analysed using a Shimadzu GCMS‐TQ8030 triple‐quadrupole GC–MS/MS, with synthetic DFRC products used to generate a calibration curve.

### 4.8 2D‐NMR assay of lignin composition

Whole cell wall ball‐milled samples (50 mg) and enzyme lignin samples (50 mg) were prepared for 2D HSQC‐NMR in DMSO‐d_6_/pyridine‐d_5_ (Kim & Ralph, 2010) on a Bruker Biospin Avance 700 MHz NMR. The DMSO solvent peak was used as an internal reference (*δ*_c_ 39.52, *δ*_H_ 2.5 ppm).

### 4.9 Measurement of breaking force

The stem strength was measured using plant lodging tester YYD‐1 (Hangzhou TOP Instrument Co., Ltd. Hangzhou, China) at mature stage according to the methods of (Luo et al., 2019) as follows. The mature stem was placed on the groove of support pillars with a distance of 10 cm. Aligning the centre position of the stem to the pressure probe. The breaking strength was the peak value when the stem was broken off and its unit was Newtons (N).

### 4.10 Scanning electron microscopic (SEM) observation

The last branch on the top at mature stage from WT and KO‐7 was sliced by hand with same thickness and the cross section first treated with 0.5 M NaOH for 2 hr or 1% H_2_SO_4_ for 1 hr, followed by washing with distilled water until reaching pH 7.0. The cross sections were frozen in liquid nitrogen and observed by S‐3400 N SEM (Hitachi) at an accelerating voltage of 2 kV.

### 4.11 In vitro assays for antifungal activity

To determine the resistance of oilseed rape against *S. sclerotiorum*, 12 detached leaves from the WT and *f5h* mutant lines both at the five‐leaf stage were inoculated, and lesion area was measured. Twelve stems at flowering and maturation time of the WT and the *f5h* mutant lines were inoculated with *S. sclerotiorum*. All tests were repeated three times. Both the leaf and stem were inoculated with two inocula, and the *S. sclerotiorum* resistance test is as follows.

The maintained *S. sclerotiorum* isolate from (Mei, Qian, Disi, Yang, & Qian, 2010) was cultured on potato dextrose agar (PDA) medium (20% potato, 2% dextrose and 1.5% agar) plates at 22°C in dark, and the 6‐mm‐diameter mycelia agar plugs punched from the growing margin of 3‐day‐old culture of *S. sclerotiorum* placed on the detached leaves or wounded stems gently. The plant *S. sclerotiorum* resistance was evaluated according to (Mei, Wei, Disi, Ding, & Wei, 2012) with little change. Briefly, the stems from flowering stage or mature stage were cut at the height of 10 cm from the ground, and the two ends were wrapped with polyethylene film to maintain freshness. Two separate wounds on the stem artificially made by 4‐mm‐diameter puncher were inoculated with the prepared inoculum. The petiole cut site of the detached leaf was wrapped with polyethylene film to keep the fresh. The inoculated leaves and stems were placed in a plastic box, which was covered with moist towels and filter paper in the bottom. To keep the moisture in the box, the top was covered and sealed by polyethylene film. The plastic box was kept in incubator at 22°C in dark. The leaf lesion area and stem lesion length were recorded 2 and 3 days after inoculation, respectively.

## 5 STATISTICAL ANALYSIS

For multiple comparison, significance analysis was performed with one‐way ANOVA followed by Tukey's post‐hoc tests. Statistical analysis was performed using SPSS Statistics software (version 13.0). Details of statistical results are in Table S3.

## CONFLICTS OF INTEREST

The authors declare no conflict of interest.

## AUTHOR CONTRIBUTIONS

Liezhao Liu and Jiana Li conceived the study. Cunmin Qu performed the fieldwork. Xueping Chen constructed the vector and performed genetic transformation. John Ralph and Rebecca A. Smith performed the DFRC lignin monomer and NMR analyses and contributed to manuscript preparation. Xingying Yan and Shuyao Ran performed anatomical analysis. Yanru Cao performed the phenotype test, data analysis, and wrote the manuscript. All authors read and approved the final manuscript.

## Supporting information


**Figure S1.** Targeted mutagenesis of *F5H* in *B. napus* is identified by sequencing KO‐8 plants

**Figure S2.** Targeted mutagenesis of *F5H* in *B. napus* is identified by sequencing KO‐10 plants

**Figure S3.** Sanger sequence of C base insertion of the target region

**Figure S4.** Sanger sequence of A base insertion of the target region

**Figure S5.** Resistance of KO‐7 to *S. sclerotiorum* infections at the flowering stage

**Table S1.** Primers used in this study

**Table S2.** Lignin content and monolignol composition of cell walls from the stems of WT and KO‐7 plants

**Table S3.** Statistical results of breaking force, lesion area and lesion length

## Data Availability

The data that support the finding of this study are available in the supplementary materials of this article.
