# Supplementary figures and images for: Knockout of the lignin pathway gene BnF5H decreases the S/G lignin compositional ratio and improves Sclerotinia sclerotiorum resistance in Brassica napus
Source: Plant Cell Environ. 2021 Dec 1;45(1):248–61. doi: 10.1111/pce.14208 (PMC9084453; doi:10.1111/pce.14208)

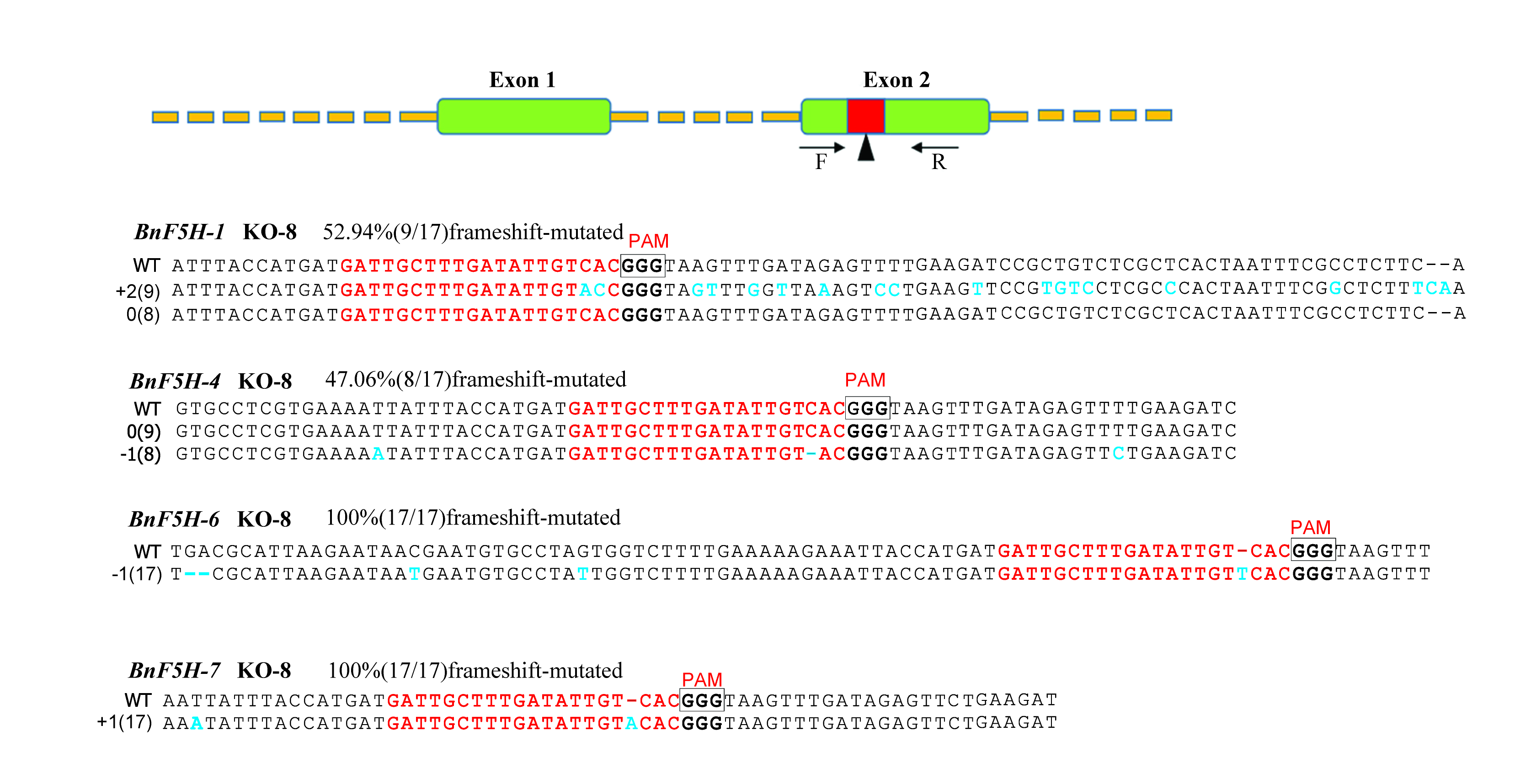

Supplement: Supplementary file 1 — Figure S1. Targeted mutagenesis of F5H in B. napus is identified by sequencing KO‐8 plants [file PCE-45-248-s005.tif]

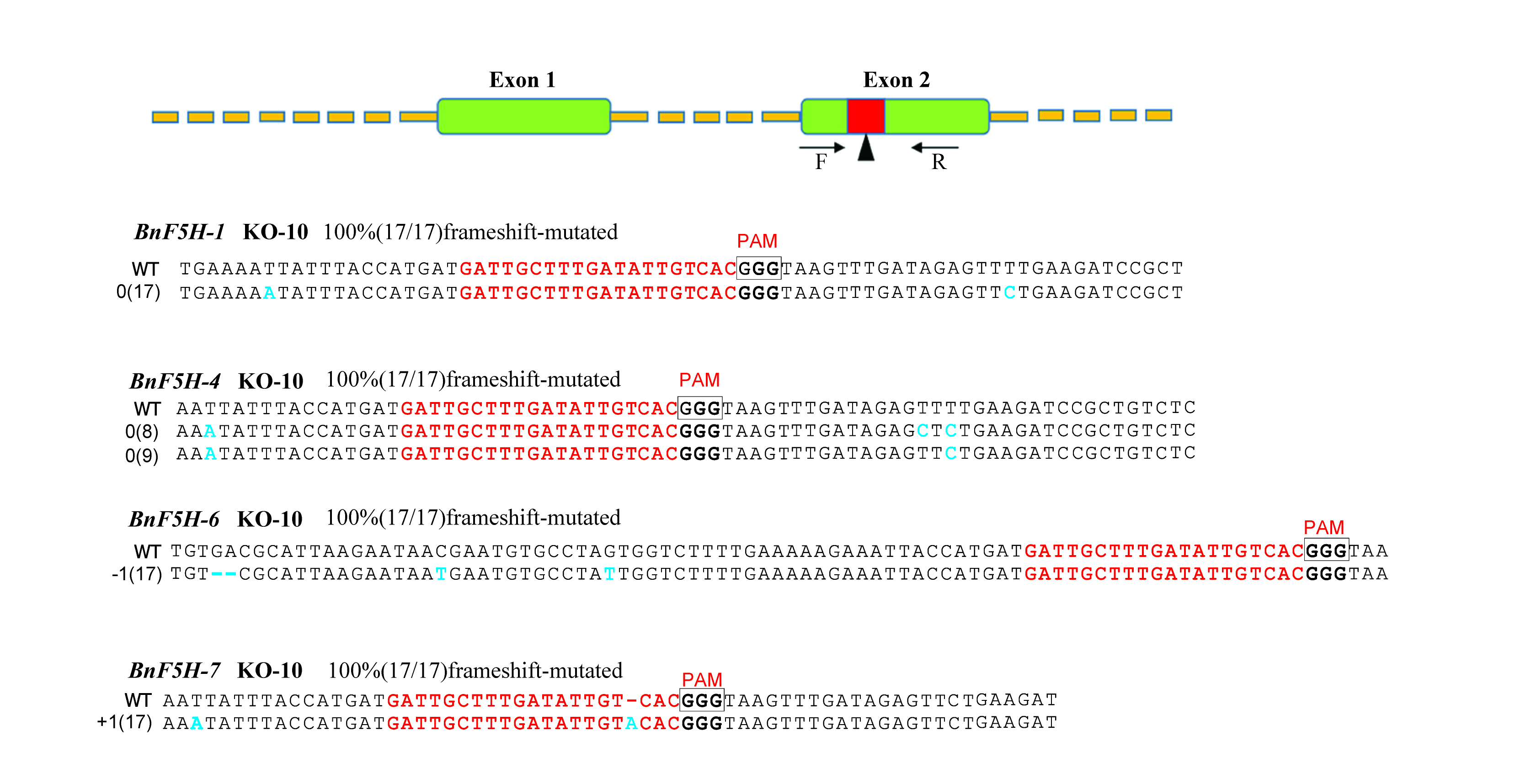

Supplement: Supplementary file 2 — Figure S2. Targeted mutagenesis of F5H in B. napus is identified by sequencing KO‐10 plants [file PCE-45-248-s008.tif]

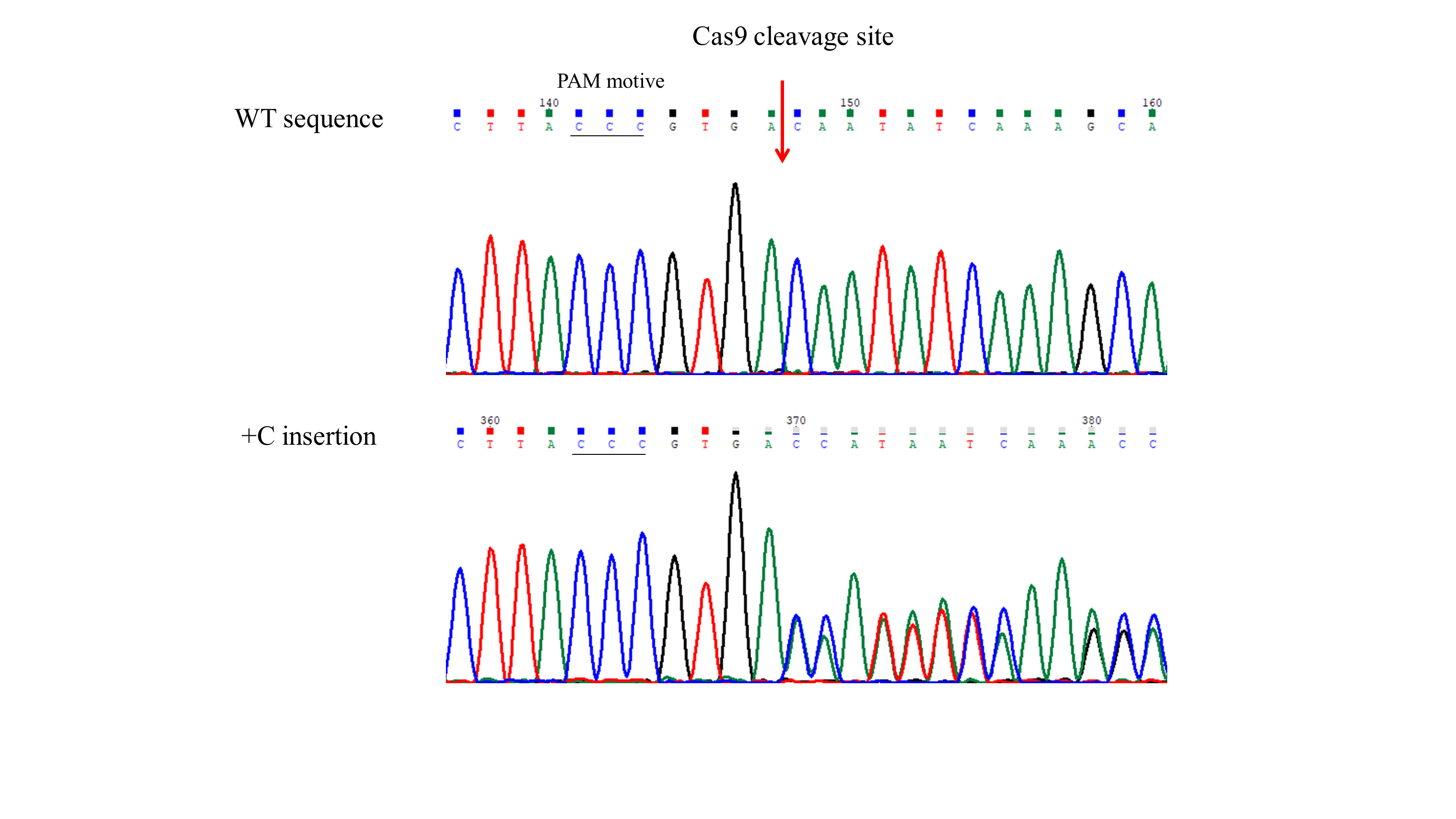

Supplement: Supplementary file 3 — Figure S3. Sanger sequence of C base insertion of the target region [file PCE-45-248-s002.tif]

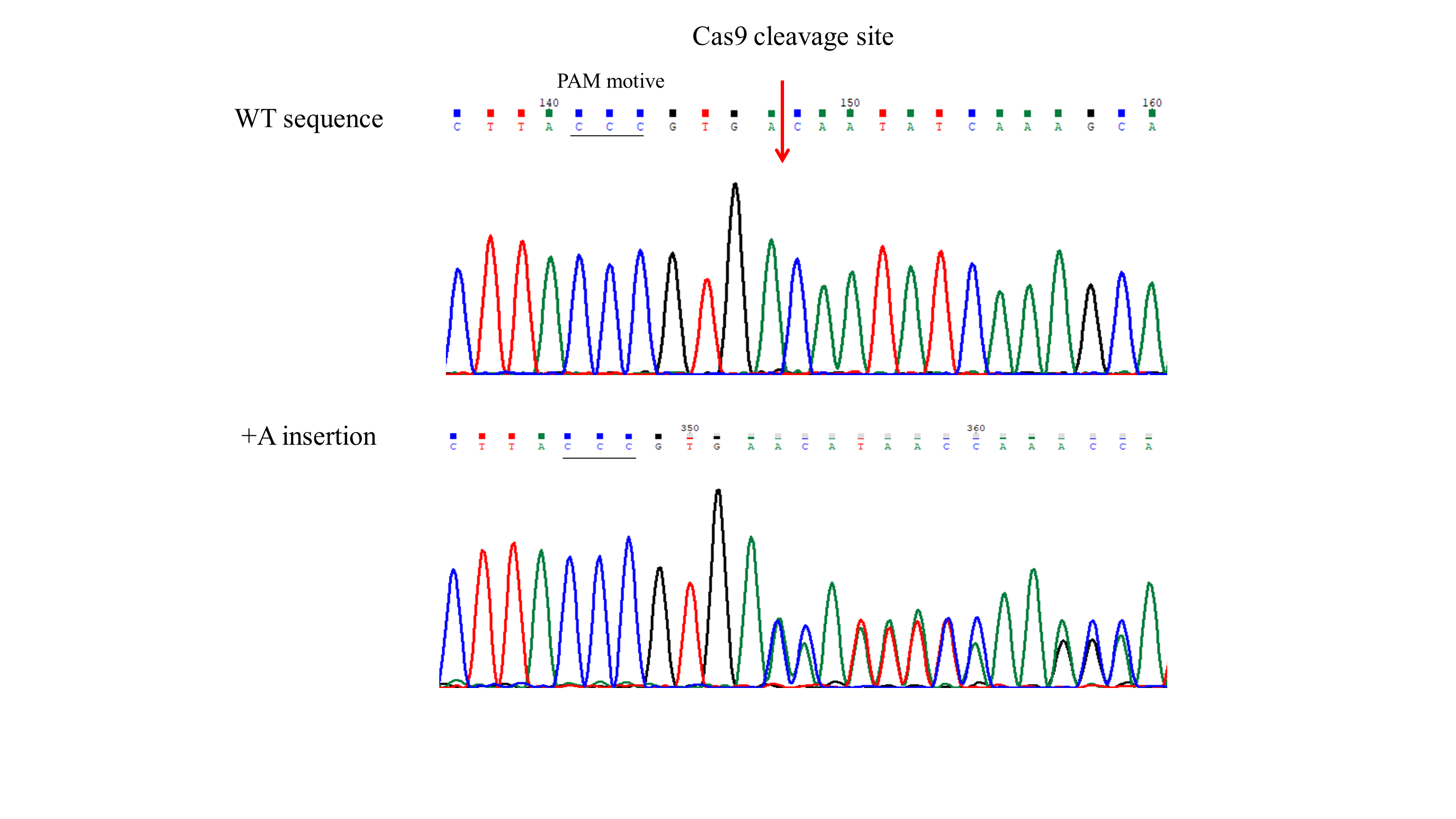

Supplement: Supplementary file 4 — Figure S4. Sanger sequence of A base insertion of the target region [file PCE-45-248-s006.tif]

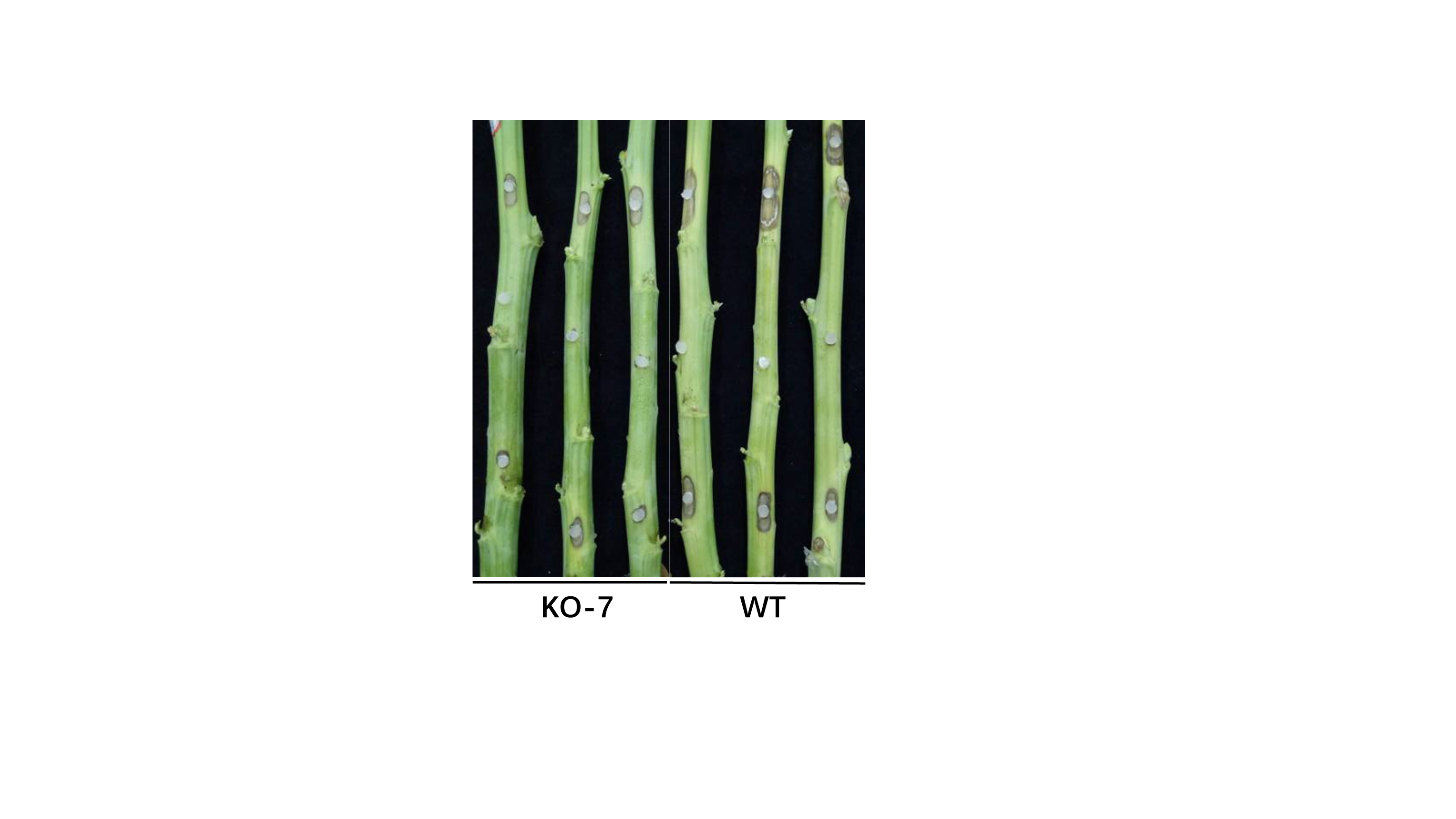

Supplement: Supplementary file 5 — Figure S5. Resistance of KO‐7 to S. sclerotiorum infections at the flowering stage [file PCE-45-248-s003.tif]
